# Experimental transmission of West Nile Virus and Rift Valley Fever Virus by *Culex pipiens* from Lebanon

**DOI:** 10.1371/journal.pntd.0005983

**Published:** 2018-01-11

**Authors:** Renée Zakhia, Laurence Mousson, Marie Vazeille, Nabil Haddad, Anna-Bella Failloux

**Affiliations:** 1 Lebanese University, Faculty of Public Health-II, Laboratory of Immunology and Vector Borne Diseases, Fanar, Lebanon; 2 Institut Pasteur, Department of Virology, Arboviruses and Insect Vectors, Paris cedex 15, France; Aix Marseille University, Institute of Research for Development, and EHESP School of Public Health, FRANCE

## Abstract

West Nile virus (WNV) and Rift Valley fever virus (RVFV) are two emerging arboviruses transmitted by *Culex pipiens* species that includes two biotypes: *pipiens* and *molestus*. In Lebanon, human cases caused by WNV and RVFV have never been reported. However, the introduction of these viruses in the country is likely to occur through the migratory birds and animal trades. In this study, we evaluated the ability of *Cx*. *pipiens*, a predominant mosquito species in urban and rural regions in Lebanon, to transmit WNV and RVFV. *Culex* egg rafts were collected in the West Bekaa district, east of Lebanon and adult females of *Cx*. *pipiens* were experimentally infected with WNV and RVFV Clone 13 strain at titers of 1.6×10^8^ and 1.33×10^7^ plaque forming units (PFU)/mL, respectively. We estimated viral infection, dissemination and transmission at 3, 7, 14 and 19 days post infection (dpi). Results showed that infection was higher for WNV than for RVFV from 3 dpi to 19 dpi. Viral dissemination and transmission started from 3 dpi for WNV; and only from 19 dpi for RVFV. Moreover, *Cx*. *pipiens* were able to excrete in saliva a higher number of viral particles of WNV (1028 ± 405 PFU/saliva at 19 dpi) than RVFV (42 PFU/saliva at 19 dpi). *Cx*. *pipiens* from Lebanon are efficient experimental vectors of WNV and to a lower extent, RVFV. These findings should stimulate local authorities to establish an active entomological surveillance in addition to animal surveys for both viruses in the country.

## Author summary

West Nile virus (WNV) and Rift Valley fever virus (RVFV) are two emerging mosquito-borne arboviruses mainly transmitted by *Culex* mosquitoes. WNV considered one of the most important causative agent of viral encephalitis has a wide distribution in many tropical and temperate countries including the Middle East. RVFV is mainly distributed in Sub-Saharan Africa but epizootics were also reported in Egypt, Saudi Arabia and Yemen. The mosquito vector belongs to the *Culex pipiens* species which includes two biotypes: *pipiens* and *molestus*. Both biotypes are the most widely distributed mosquitoes in Lebanon. Using experimental infections of mosquitoes, our study showed that *Cx*. *pipiens* populations collected in West Bekaa were susceptible to infection by these two viruses and ensured efficient transmission of WNV and to a lesser extent, RVFV. Our findings may help to prepare a control strategy more adapted to these mosquito vectors.

## Introduction

West Nile virus (WNV) and Rift Valley fever virus (RVFV) are two important emerging mosquito-borne zoonotic agents transmitted by *Culex pipiens*, a complex of sibling species that includes *Cx*. *pipiens s*.*s*., *Cx*. *quinquefasciatus* and possibly *Cx*. *australicus* [1, 2]. *Cx*. *pipiens s*.*s*. includes two biotypes or subspecies: *Cx*. *pipiens pipiens* and *Cx*. *pipiens molestus* [1, 3]. The first biotype is primarily a bird-feeding mosquito present in temperate areas while *Cx*. *pipiens* biotype *molestus* feeds on mammals (mainly human) and thrives in sewers in temperate and sub-tropical regions [3, 4]. Because morphological identification of these biotypes is not possible, they can only be distinguished using molecular techniques [3–5].

WNV is a member of the Flavivirus genus (Flaviviridae family) and was first isolated in Uganda in 1937 [6]. Usually, only 20% of infected individuals develop symptoms and less than 1% of infected people develop serious and potentially fatal neurological illnesses such as meningitis and encephalitis. Birds are considered the main reservoir of the virus and *Cx*. *pipiens* is recognized as one of the primary enzootic vector [7]. WNV infections have been reported in many tropical and temperate countries in Africa, Europe, Asia and America. The Middle East and North Africa (MENA) region has been long considered as a WNV-endemic area [8, 9]. Locally acquired cases have been recently reported in Israel [10], Greece [11], Turkey [12], and Italy [13]. In addition, evidence of WNV circulation has been reported in Jordan [14] and Egypt [15]. The introduction of WNV into the United States in 1999, which constitutes a turning point in WNV epidemiology, is thought to have originated from Israel following introduction from Africa [16, 17].

RVFV belongs to Phlebovirus genus (Bunyaviridae family). It was first identified in Kenya in 1931 [18]. This virus usually affects livestock and causes abortion. The main enzootic vectors belong to the *Aedes* genus [19]. However, several *Culex* species, including *Cx*. *pipiens* are considered secondary vectors and contribute to the transmission of RVFV to humans [19]. Infected people can be asymptomatic or develop a mild febrile disease. In less than 10% of cases, people may develop more severe symptoms such as encephalitis and hemorrhagic fever. RVFV was responsible for numerous outbreaks among animals and humans in Sub-Saharan Africa [20] up to Mauritania [21] but also in Egypt [22]. In the Middle East, epizootics were reported in Saudi Arabia and Yemen [23].

In Lebanon, *Cx*. *pipiens* is a predominant mosquito species besides another vector of arboviruses, *Aedes albopictus* [24, 25]. *Cx*. *pipiens* colonizes urban and rural habitats whereas *Ae*. *albopictus* is mostly present in the densely populated coastal fringe. Local *Ae*. *albopictus* are competent to transmit Chikungunya virus and to a lesser extent, Dengue virus [26]. The vector competence of local populations of *Cx*. *pipiens* to transmit WNV and RVFV has never been evaluated. Diseases caused by these two viruses have never been reported in Lebanon. Nevertheless, a serological study conducted in a main hospital in the capital city of Beirut, confirmed the presence of neutralizing WNV antibodies in blood donors [27]. In fact, Lebanon is situated in a WNV-endemic area and located on the flyways of migratory birds with potential introduction of the virus into the country. Moreover, Lebanon is geographically close to Yemen and Saudi Arabia, regions where RVFV had circulated actively. Intensive livestock trade between Lebanon and these countries increases the risk of RVFV introduction.

Here, we assess the vector competence of local populations of *Cx*. *pipiens* towards WNV and RVFV. We estimate viral infection, dissemination and transmission at different days after experimental infections.

## Materials and methods

### Mosquito collections

**Fig 1:**
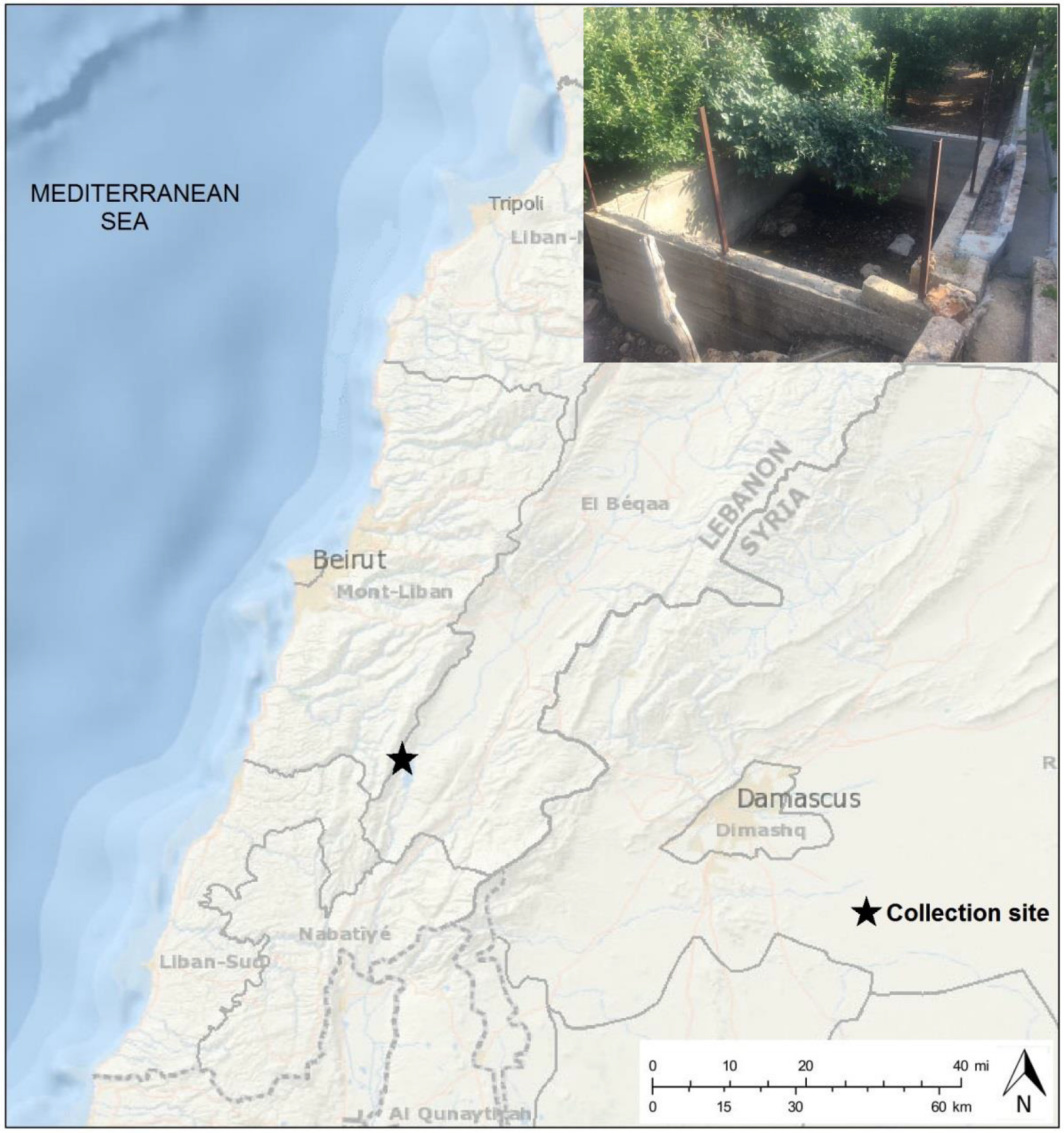
Map showing the geographic location of Lebanon East of the Mediterranean basin. This map indicates the site of mosquito egg collections in West Bekaa region. The map was modified using PowerPoint from http://landsatlook.usgs.gov/ (Credit: U.S. Geological Survey Department of the Interior/USGS—U.S. Geological Survey). Photo: breeding site (N. Haddad).

*Culex* egg rafts were sampled in June 2015 in Bab Mareh, in the West Bekaa district, a sub-humid, agricultural area in east of Lebanon with large stagnant water systems (Fig 1). Egg rafts were collected on the water surface in an artificial basin and placed individually in a tube containing 30 mL of water collected from the breeding site.

### Mosquito preparation for vector competence study

Collected egg rafts were shipped to the Laboratory of Arboviruses and Insect Vectors (AIV) at the Institut Pasteur, Paris. They were reared until the adult stage. Adults emerging from each raft were morphologically identified and only *Cx*. *pipiens* species were retained for this study.

### Viruses and blood meal preparation

Two viruses were used in this study: WNV lineage 1 strain isolated from a horse in Camargue (France) in 2000 [9] and an avirulent RVFV strain Clone 13 isolated from a human case in Bangui (Central African Republic) in 1974 [28]. After passages on Vero (E6) cells (ATCC cell lines), both viruses were produced on C6/36 mosquito cells. Viral stocks were stored at -80°C until use. The infectious blood meal was composed of a viral suspension (1:3) diluted in washed rabbit erythrocytes (New Zealand White rabbit, Charles River) collected at the day of mosquitoes infection. A phagostimulant (ATP) was added at a final concentration of 5 mM. Virus titer in the blood meal was 1.6×10^8^ plaque forming units (PFU)/mL for WNV and 1.33×10^7^ PFU/mL for RVFV.

### Artificial feeding of female mosquitoes

The susceptibility of Lebanese *Cx*. *pipiens* mosquitoes to WNV and RVFV was tested on F0 and F1 generation respectively. Ten-to-twelve day-old female *Cx*. *pipiens* mosquitoes were left to starve for 48 h in Biosafety Level 3 (BSL3) insectary at 28±1°C with 80% relative humidity and a 16h:8h photoperiod. Females were then allowed to feed for one hour through a chicken skin membrane (obtained from a commercially purchased chicken) covering the base of a capsule of the feeding system (Hemotek) containing the blood-virus mixture maintained at 37°C. Fully engorged females were sorted, then transferred in cardboard containers and maintained with 10% sucrose at 28±1°C until examination.

### Saliva collection

Around 20 female mosquitoes were tested at 3, 7, 14 and 19 days post-infection (dpi). For each mosquito, saliva was collected using the forced salivation technique [29]. Briefly, mosquitoes were chilled, their legs and wings removed and the proboscis was inserted into 20 μL tip filled with 5 μL of Fetal Bovine Serum (FBS). After 45 min, medium containing the saliva was expelled into 0.2 mL tube containing 45 μL of Dulbecco’s MEM (DMEM) medium. Collected saliva and the remaining mosquito bodies were conserved at -80°C for further analysis.

### Viral detection in mosquitoes

In order to assess the ability of both viruses to invade and cross the midgut barrier, the infection rate (IR) and the dissemination efficiency (DE) were determined. IR reflects the proportion of female mosquitoes with infected bodies (thorax and abdomen including the midgut) among tested specimens while DE is the proportion of female mosquitoes with infected head (detection of the virus having succeeded to reach the mosquito general cavity) among tested ones. Thus, heads and bodies were separated and ground each in 300 μL DMEM supplemented with 3% FBS. After centrifugation, the supernatant of each homogenate was conserved at -80°C. Then, 20 μL of each sample were diluted in 180 μL DMEM supplemented with 2% FBS and distributed in serial dilutions from 10^−1^ to 10^−3^ in duplicates on Vero cell monolayers (3.10^5^ cells/well) in 96-well plates. After incubation at 37°C for 6 days, inoculum was removed and the cells were fixed and stained using a crystal violet solution (0.2% in 10% formaldehyde and 20% ethanol). After washing, the presence or absence of cytopathic effect was noted.

The capacity of the WNV and RVFV to cross the salivary glands barrier was evaluated by determining the transmission efficiency (TE) which corresponds to the proportion of female mosquitoes that secrete infectious saliva among tested specimens. The number of infectious particles within collected saliva samples was estimated on Vero cell culture and expressed as PFU/saliva. Briefly, 20 μL of each saliva were diluted in 280 μL DMEM 2% FBS. The total volume was inoculated on a monolayer of Vero cells (8.10^5^cells/well) in six-well plates. Cells were incubated at 37°C for 6 days under an overlay consisting of DMEM, 2% FBS, 1% antibiotic-antimycotic mix and 1% agarose. The lytic plaques were counted after staining with a crystal violet solution.

### Statistical analysis

Proportions (IR, DE and TE) were compared using Fisher’s exact test and sample distributions (number of viral particles) with the Kruskal-Wallis test. Statistical analyses were conducted using the Stata software (StataCorp LP, Texas, and USA). P-values<0.05 were considered significant.

## Results

### Artificial feeding

Collected egg rafts were hatched in laboratory conditions and provided 480 adult females of *Culex pipiens* (F0 generation); of those only 174 (36.25%) had successfully fed on a WNV-infected blood. A batch of 600 F1 female mosquitoes was used for the RVFV infection assay. Of those, only 91 (15.16%) had successfully fed on infected blood.

### Viral infection

**Fig 2:**
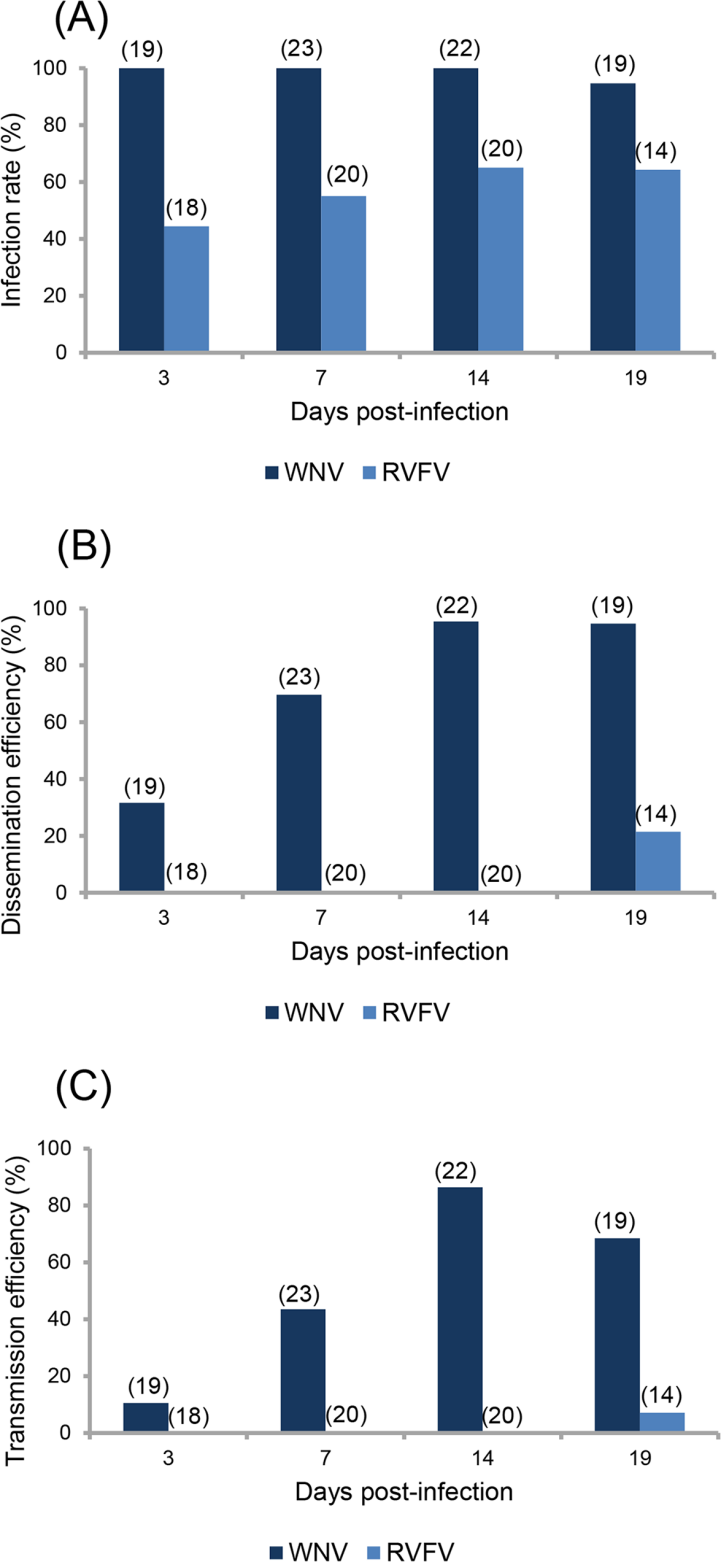
**Infection (A), Dissemination (B) and Transmission (C) of *Culex pipiens* at different days after ingestion of an infectious blood meal containing WNV or RVFV.** *Culex pipiens* F0/F1 mosquitoes were orally challenged with WNV at a titer of 1.6×10^8^ PFU/mL and RVFV at titer of 1.33×10^7^ PFU/mL using an artificial feeding system (Hemotek). At 3, 7, 14, and 19 dpi, homogenates of mosquito bodies and heads, and saliva collected from females were tested for the presence of virus on Vero cells to estimate infection rate, dissemination efficiency, and transmission efficiency, respectively. In brackets, the number of tested mosquitoes.

Infection rate (IR) for each virus was estimated by determining the number of infected bodies (abdomen and thorax) among all engorged female mosquitoes examined at each dpi (3, 7, 14 and 19) (Fig 2A). For WNV, IRs were very high (94.7–100%) from 3 to 19 dpi. For RVFV, IRs were much lower: at 3 dpi, the IR was 44.0% and increased gradually to reach 65.0% at 14 dpi and 64.3 at 19 dpi.

### Viral dissemination

The detection of viral particles in mosquito heads allowed estimating the ability of the virus to disseminate from the midgut to internal organs. For WNV, dissemination efficiency (DE) increased from 31.6% (3 dpi) to 94.7% (19 dpi) (Fig 2B). For RVFV, virus was only detected at 19 dpi with a DE of 21.4% (Fig 2B).

### Viral transmission

The ability of mosquitoes to transmit the virus was measured by detecting viral particles in saliva expectorated by mosquitoes. With WNV, transmission efficiencies were much higher than with RVFV (Fig 2C). TE increased gradually from 10.5% at 3 dpi to 68.4% at 19 dpi (Fisher’s exact test: p < 10^−4^). To note, TE decreased slightly but not significantly from 86.4% at 14 dpi to 68.4% at 19 dpi (Fisher’s exact test: p = 0.17). For RVFV, viral particles were only detected in saliva at 19 dpi with a TE of 7.1% (Fig 2C).

### Intensity of transmission

**Fig 3:**
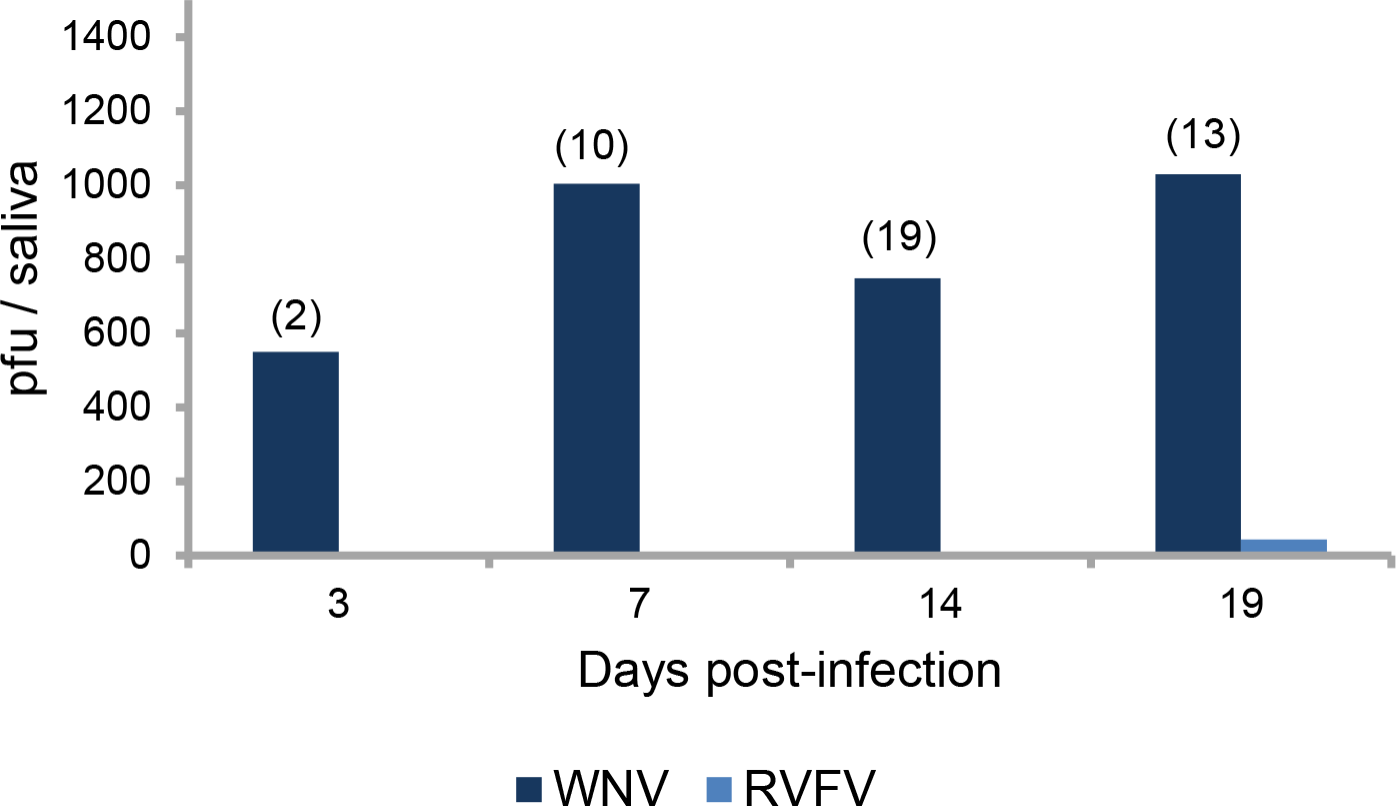
Mean titer of infectious viral particles secreted in saliva of *Culex pipiens* at different days after ingestion of an infectious blood meal containing WNV or RVFV. The number of infectious particles in saliva was estimated by inoculation of collected saliva on Vero cells and expressed as pfu/saliva. In brackets, the number of tested mosquitoes.

Mosquitoes were able to deliver an average of 550 (±450) PFU/saliva at 3 dpi with WNV, which increased to reach 1004 (±442) PFU/saliva at 7 dpi (Fig 3). Despite a decrease at 14 dpi, the viral load remained high at 19 dpi with 1028 (±405) PFU/saliva. For RVFV, only one female had infectious particles in saliva at 19 dpi with a viral load of 42 PFU (Fig 3).

## Discussion

*Culex pipiens* is the most widely distributed mosquito species in Lebanon and is suspected to transmit WNV and RVFV in several countries [7, 19]. Using experimental infections, we showed that *Cx*. *pipiens* populations collected from West Bekaa, Lebanon were susceptible to infection by these two viruses and ensured efficient transmission of WNV and to a lesser extent, RVFV.

*Cx*. *pipiens* was capable to ensure viral infection, dissemination and transmission starting from 3 dpi. Most mosquitoes exposed to the infectious blood-meal were infected as IRs reached 100% at the 4 dpi examined (3, 7, 14 and 19 dpi). Dissemination and transmission were slightly lower suggesting that not all infected mosquitoes were able to transmit WNV. Mosquitoes delivered more than 500 viral particles in saliva from 3 dpi. On the other side, infections with RVFV present different patterns: lower IR, DE and TE. Only 21% of mosquitoes were able to ensure viral dissemination at 19 dpi and 7% were able to transmit at 19 dpi. This suggests a significant role of the midgut and the salivary glands as respective barriers to the release of viruses into the body cavity and their excretion in saliva [30]. In this manner, *Cx*. *pipiens* was less susceptible to RVFV than to WNV.

Overall, *Cx*. *pipiens* can transmit experimentally both viruses but the time interval between the ingestion of the viremic blood-meal and the ability to transmit the virus termed the extrinsic incubation period (EIP) was 3 days for WNV. For RVFV, we only found one mosquito able to transmit the virus 19 days after ingestion. It is likely that more females would have been able to transmit the virus if more mosquitoes were able to feed on RVFV-infected blood. *Cx*. *pipiens* from Tunisia showed similar EIP of 3 days with WNV and a much shorter EIP of 3 days with RVFV [31] underlining the significant role of mosquito genotype in specific interactions between mosquito and virus genotypes; these interactions promoting adaptation of viral lineages to specific mosquito vector genotypes influence the outcome of transmission [32]. In addition, when viral dose increases in blood meals, transmission efficiency also increases suggesting that hosts presenting a high viremia may infect more mosquitoes [33]. Animals susceptible to RVFV can develop very high viremia, higher than 10^10.1^ MIPLD50 (mouse intraperitoneal 50% lethal dose/mL) in lambs [34]. Then, at higher titers of blood meal, RVFV may infect more mosquitoes.

In conclusion, the predominant *Cx*. *pipiens* mosquito in Lebanon is susceptible to both viruses, WNV and RVFV. As Lebanon is located in a region where WNV and RVFV can be potentially introduced (respectively through migratory birds and animal trades), local health authorities should establish an active surveillance to detect any new human cases in addition to reinforce the entomological surveillance allowing an early viral detection in field-collected mosquitoes.
